# Field-Effect Transistor Based on 2D Microcrystalline MoS_2_ Film Grown by Sulfurization of Atomically Layer Deposited MoO_3_

**DOI:** 10.3390/nano12193262

**Published:** 2022-09-20

**Authors:** Ivan V. Zabrosaev, Maxim G. Kozodaev, Roman I. Romanov, Anna G. Chernikova, Prabhash Mishra, Natalia V. Doroshina, Aleksey V. Arsenin, Valentyn S. Volkov, Alexandra A. Koroleva, Andrey M. Markeev

**Affiliations:** 1 Moscow Institute of Physics and Technology, National Research University, Institutskii per. 9, 141701 Dolgoprudny, Russia; 2 Center for Photonics & 2D Materials, Moscow Institute of Physics and Technology, National Research University, 141700 Dolgoprudny, Russia; 3 Center for Nanoscience and Nanotechnology, Jamia Millia Islamia (Central University), New Delhi 110025, India

**Keywords:** TMDC, ALD, sulfurization, microcrystalline film, Raman spectroscopy, field effect transistor

## Abstract

Atomically thin molybdenum disulfide (MoS_2_) is a promising channel material for next-generation thin-body field-effect transistors (FETs), which makes the development of methods allowing for its controllable synthesis over a large area an essential task. Currently, one of the cost-effective ways of its synthesis is the sulfurization of preliminary grown oxide- or metallic film. However, despite apparent progress in this field, the electronic quality of the obtained MoS_2_ is inferior to that of exfoliated samples, making the detailed investigation of the sulfurized films’ properties of great interest. In this work, we synthesized continuous MoS_2_ films with a thickness of ≈2.2 nm via the sulfurization of an atomic-layer-deposited MoO_3_ layer. X-ray photoelectron spectroscopy, transmission electron microscopy, and Raman spectroscopy indicated the appropriate chemical composition and microcrystalline structure of the obtained MoS_2_ films. The semiconductor quality of the synthesized films was confirmed by the fabrication of a field-effect transistor (FET) with an I_on_/I_off_ ratio of ≈40, which was limited primarily by the high contact resistance. The Schottky barrier height at the Au/MoS_2_ interface was found to be ≈1.2 eV indicating the necessity of careful contact engineering. Due to its simplicity and cost-effectiveness, such a technique of MoS_2_ synthesis still appears to be highly attractive for its applications in next-generation microelectronics. Therefore, further research of the electronic properties of films obtained via this technique is required.

## 1. Introduction

The two-dimensional (2D) Van der Waals materials, especially transition-metal dichalcogenides (TMDC), currently attract a great deal of interest for electronic applications due to their unique properties manifested at a nanoscale thickness [1,2]. In particular, they can serve as a thin channel material in next-generation FETs due to the possibility of thickness downscaling to a single monolayer (≈0.7 nm) [3]. They have also demonstrated outstanding optoelectronic, catalytic, and gas-sensing properties [4,5,6]. Recently, in addition to the novel physics of 2D TMDC materials, the issues of their synthesis also began to be widely discussed. Besides the thickness and crystalline structure control having a strong influence on the film performance [7,8], the ability of their growth over the relevant area becomes a very important task. Synthesized TMDCs’ performance is strongly affected by defects such as vacancies, impurities, and grain boundaries introduced during the fabrication process [1]. To date, several growth techniques, including direct atomic layer deposition (ALD) [9,10,11] or metal-organic chemical vapor deposition (MOCVD) [12,13,14,15,16,17] epitaxy have been suggested. In contrast to significant progress regarding the WS_2_ growth reported by Groven et al. [18], ALD-grown MoS_2_ films are typically weakly crystalline [11] due to the low process temperature (≈350–450 °C) and contain out-of-plane oriented grains with a size of ≈10–20 nm, which results in poor carrier mobility, making such films more appropriate for catalysis applications [19]. In contrast, the typical grain size for MOCVD-grown MoS_2_ was found to be significantly higher (≈0.1–1 µm) [20,21,22], and such films have shown superior electrical performance [16,22,23]. However, a strong cross-correlation between the growth process parameters is frequently reported for MOCVD, making the reproducibility quite challenging [17]. In addition, the growth process can take a long time (up to dozens of hours [15]), which slightly deteriorates the practical significance of this approach. Therefore, an alternate method, namely the sulfurization of the preliminary grown oxide or metallic seed film, has also been suggested [24,25,26,27,28]. Due to its simplicity and cost-effectiveness, it looks highly promising for further use in mass production. Furthermore, in contrast to MOCVD epitaxy, this method is much less sensitive to the substrate material, which makes it possible to synthesize MoS_2_ on arbitrary substrates. From the practical point of view, the seed molybdenum oxide film should predominantly be grown by ALD due to the perfect repeatability and thickness control at the angstrom-scale not achievable by other deposition techniques [29,30,31,32]. ALD also makes it easy to produce high-quality ternary oxides [33,34]. Typically, TMD films obtained via a sulfurization technique are microcrystalline, which makes them potentially suitable for electronic device fabrication [24,35,36,37,38,39]. Overall, despite the relatively weak gate modulation in FET structures based on such films [40,41], the consequent systematic studies aiming to improve the MoS_2_ quality itself and also decrease the contact resistance at source and drain contacts are in progress [42,43,44]. This leads to the conclusion that research on the electronic properties of microcrystalline MoS_2_ films obtained via sulfurization is of significant practical interest. Moreover, taking into account the fact that the seed film’s chemical state, microstructure, and possible impurity presence may impact the resulting 2D layer properties [45], it becomes evident that detailed experimental research is necessary in each individual case.

Despite the significant progress in this field reported by Mahlouji et al. [37,46], in their particular case, the seed MoO_3_ films were grown by plasma-assisted ALD at 50 °C. However, such a low temperature resulted in undesirable hydrogen film contamination [47]. Moreover, plasma-assisted ALD opportunities regarding the film conformality on high-aspect-ratio structures are inferior to that of conventional thermal ALD [31]. Thus, the aim of the current work was to fabricate MoS_2_ films using a cost-effective sulfurization method from seed MoO_3_ obtained via thermal ALD from the similar (N^t^Bu)_2_(NMe_2_)_2_Mo precursor and ozone at a higher temperature, as well as estimate their performance in FET structures and investigate their intrinsic properties. Taking into account the requirement of seed film continuity as well as reported optimal thickness [37], its thickness was chosen to be 2.5 nm. Considering the necessity of seed oxide characterization, the XPS analysis was carried out before and after sulfurization. Next, the obtained MoS_2_ films were used to fabricate FET structures, whose performance was subsequently evaluated and compared to that of an exfoliated MoS_2_ flake with a similar thickness. Therefore, we believe that our work can fulfill the existing gap and supplement the existing experimental data on the electronic properties of microcrystalline MoS_2_ films.

## 2. Materials and Methods

### 2.1. MoS_2_ Film Preparation and Characterization

Thin (2.5 nm) MoO_3_ films were grown in a commercially available hot-wall Picosun R100 ALD reactor at T = 250 °C. Bis(t-butylimido)bis(dimethylamino)molybdenum(VI) ((^t^BuN)_2_(NMe_2_)_2_Mo) and ozone (O_3_) were used as the precursor and reactant (oxygen source), respectively. The liquid (^t^BuN)_2_(Nme_2_)_2_Mo source was kept at 60 °C during the ALD process to acquire adequate vapor pressure. The IN-USA AC-2025 generator, operated at a total oxygen input flow of 700 sccm (backside pressure 20 psig), was used as an O_3_ supply. The estimated O_3_ concentration was approximately 60 g/Nm^3^ at actual operating conditions. The precursor and O_3_ feeding times were fixed at 1.6 s and 16 s, respectively. The ALD process started with the O_3_ exposure for 20 s to remove residual surface organic contamination. Preliminarily cleaned (piranha solution and deionized water) and annealed (1 h at 1000 °C) (0001)-oriented sapphire (Al_2_O_3_) pieces with a size of 1 × 2 cm were used as substrates for ALD growth. MoO_3_ deposition was performed in a single ALD process for all samples.

The sulfurization of grown MoO_3_ films was carried out separately for every sample in a three-zone tube furnace HZS-1200 (Carbolite Gero) equipped with a 32 mm outer diameter quartz tube. A crucible containing 500 mg of sulfur flakes was located upstream from the sample in a tube. Sulfur flakes were loaded in a boat placed at the end of the heated section of the split-tube three-zone furnace (all zones were maintained at the same temperature). After loading the samples, the tube was purged for 1 h by the 5% H_2_-Ar mixture (99.9999%) to obtain a controllable gas atmosphere (total gas flow was fixed at 150 sccm). After the purge, the tube was heated to 900 °C with a heating rate of 15 °C/min under the continuous H_2_-Ar flow (10 sccm). The sample remained for 30 min at the target temperature; afterward, the heating system was switched off, allowing the furnace to cool down.

The chemical state and composition of the obtained MoS_2_ film were analyzed by XPS using a Theta Probe spectrometer under high-vacuum conditions (base pressure < 2 × 10^−9^ mbar) with a monochromatic Al-Kα X-ray source (1486.6 eV). Photoelectron spectra were acquired using fixed analyzer transmission (FAT) mode with 50 eV pass energy. Angle-resolved spectra were collected within an angular range (θ) of 25–75° in order to estimate the film thickness. Atomic force microscopy of the MoS_2_ film (AFM, NT-MDT Solver-Pro tool) was performed in semi-contact mode using a silicon tip with a radius < 10 nm (HA-NC, SCANSENS). The film crystalline structure was studied by transmission electron microscopy (TEM, FEI Tecnai G2) at an accelerating voltage of 200 kV. Raman spectroscopy was also used to confirm the MoS_2_ film structure and thickness. A LabRAM Evolution (Horiba Scientific) instrument with a 632.8 nm laser source and a 1 cm^−1^ spectral resolution was used to perform the spectral measurements. A diffraction grating of 1800 lines/mm and a ×100 objective lens (numerical aperture = 0.90) was utilized in these experiments. The laser spot diameter was 0.45 µm. All peaks were fitted with Lorentzian functions.

### 2.2. FET Preparation and Characterization

**Figure 1:**
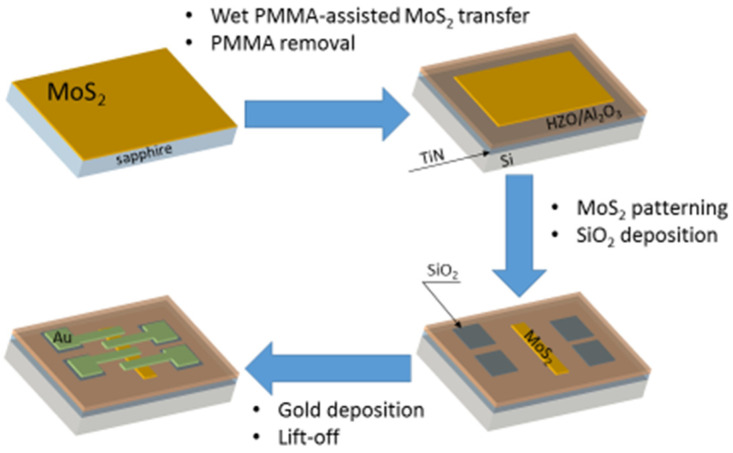
The process flow schematic of FET structure fabrication.

In order to investigate the electrical properties of the synthesized MoS_2_ films, three bottom-gate FET structures were fabricated from films obtained in separate sulfurization processes. First, the MoS_2_ films were transferred from sapphire onto the target Si (n-type)/TiN (50 nm)/Hf_0.5_Zr_0.5_O_2_ (10 nm)/Al_2_O_3_ (5 nm) substrates, which were preliminarily annealed at 400 °C for Hf_0.5_Zr_0.5_O_2_ layer crystallization. The PMMA film coating was used during the transfer process. After PMMA removal in acetone and MoS_2_ channel patterning by dry etching, the source and drain contact pad areas were covered by isolating the SiO_2_ layer using the lift-off technique. Before each lithography process, the samples were soft annealed at 200 °C for 2 h in a medium vacuum under nitrogen flow. Finally, the source, drain, and gate contacts were formed by electron-beam lithography and subsequent metal deposition (50 nm Au). The process flow schematic is shown in Figure 1. 

A similar FET structure based on an exfoliated MoS_2_ flake was also prepared as a reference device. For transfer length method (TLM) measurements, four evenly spaced contacts to the MoS_2_ channel were patterned. Electrical characterization was carried out using the Keysight B1500A measuring unit, connected to a Cascade Microtech Summit probe station.

## 3. Results and Discussion

**Figure 2:**
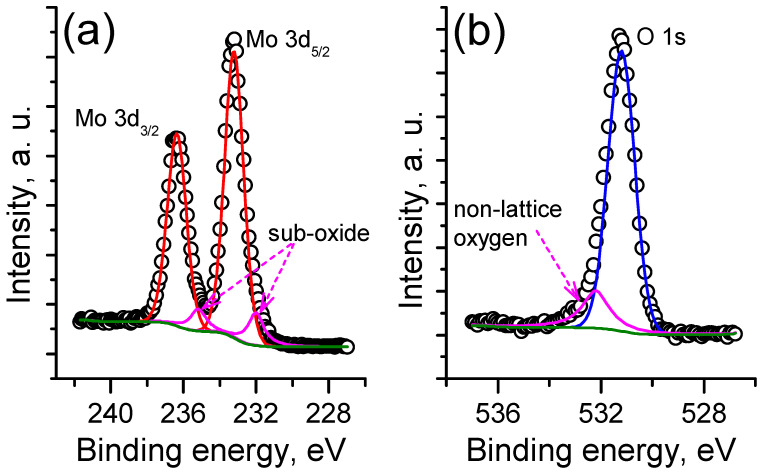
Mo3d (**a**) and O1s (**b**) XPS spectra of the initial MoO_3_ film.

First, the chemical state and the thickness of the grown MoO_3_ film were investigated by XPS. In order to avoid surface contamination, the sample was in situ transferred to an XPS analysis chamber immediately after ALD growth. The collected Mo3d and O1s XPS spectra are presented in Figure 2. The Mo3d spectrum is well described by two doublets, corresponding to the Mo^6+^ and Mo^5+^ oxidation states (Figure 2a) with binding energies of Mo3d_5/2_ line 233.2 eV and 232.0 eV, respectively. This observation clearly indicates the slight oxygen deficiency in the grown film, since the non-lattice component can be found in the O1s spectrum (Figure 2b), and the VB spectrum demonstrates a visible peak in the bandgap (not shown), corresponding to the oxygen vacancy presence [47,48].The calculated O/Mo ratio was found to be ≈2.9. Since the carbon and nitrogen content in the grown film was found to be below the XPS detection limit, the observed oxygen deficiency can be eliminated by longer O_3_ exposure during growth. However, since the subsequent sulfurization step takes place in a hydrogen-containing atmosphere, this can be neglected.

**Figure 3:**
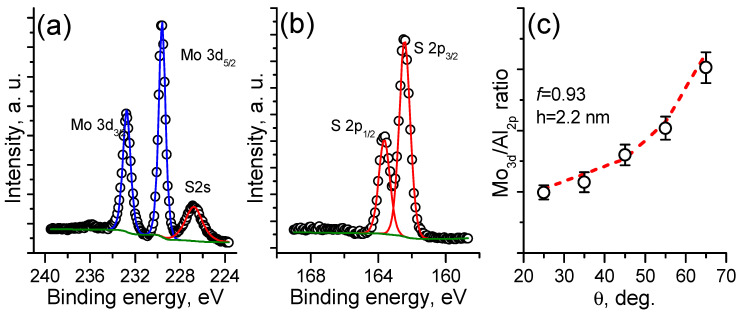
XPS spectra of Mo3d (**a**) and S2p (**b**) lines measured from the sulfurized MoO_3_ film; Mo3d/Al2p signal ratio as a function of the electron emission angle Θ (**c**).

The XPS analysis carried out immediately after sulfurization (Figure 3a,b) revealed that the Mo3d spectrum is described by a single doublet with the binding energy of the Mo3d_5/2_ line of approximately 229.6 eV, which corresponds to the Mo^4+^ state in MoS_2_ [27]. In addition, no peaks corresponding to the state of Mo being bonded with oxygen ions are observed, suggesting the complete conversion of the MoO_3_ to Mo-sulfide. The corresponding S2p spectrum consists of a single doublet with the binding energy of the S2p_3/2_ line at 162.4 eV, which coincides with the stoichiometric MoS_2_. The quantitative XPS analysis based on Mo3d and S2p sensitivity factors showed that the film stoichiometry was S/Mo ≈ 2.1 for the given sulfurization conditions. In order to estimate the obtained MoS_2_ thickness and continuity, the XPS spectra corresponding to the film and substrate were collected in the angle-resolved mode (Figure 3c). According to our previous work, such a procedure possesses sufficient accuracy [31]. It was found that the best fitting experimental data correspond to a continuous film (f ≈ 0.93) with a thickness of ≈2.2 nm (3–4 MoS_2_ monolayers). 

**Figure 4:**
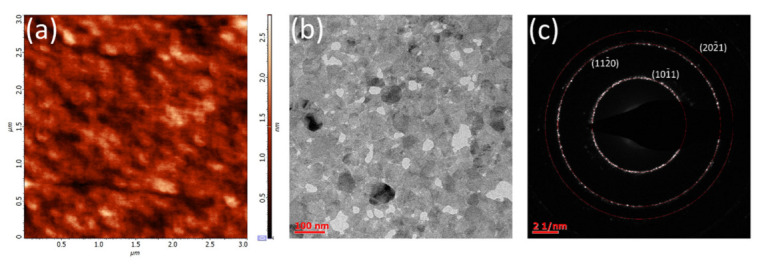
AFM (**a**) and plan-view TEM (**b**) images and selected-area electron diffraction (SAED) pattern (**c**) of MoS_2_ film, obtained via MoO_3_ sulfurization.

Next, the sulfurized film was investigated by atomic force microscopy (AFM) and transmission electron microscopy (TEM). It was found that the obtained MoS_2_ film was quite smooth, with the measured root-mean-square value of only 0.33 nm (Figure 4a), which is significantly lower than the value obtained in our previous work [28]. Furthermore, the film was found to be continuous and uniform since crystalline grains were not resolved. For the TEM analysis, the MoS_2_ film was transferred from the sapphire to a copper grid. The obtained plan-view TEM image is presented in Figure 4b.

The TEM analysis confirmed the polycrystalline nature of the obtained MoS_2_ film, which consists of grains with sizes in the range of 20–100 nm. However, the weakness of the bonds between them caused the loss of some grains during the film transfer, which is, apparently, the main reason for the observed local discontinuity. The basal planes of the grains seem to be aligned to the substrate surface, while no signs of the edge-terminated structure are observed. Obviously, the related SAED image (Figure 4c) contains only dot-like (10–11) and (11–20) diffraction rings. The absence of the diffraction ring corresponding to the (0001) plane of MoS_2_ confirms that all observed grains have their [0001] direction aligned with the out-of-plane direction, while their in-plane orientations are almost random. 

**Figure 5:**
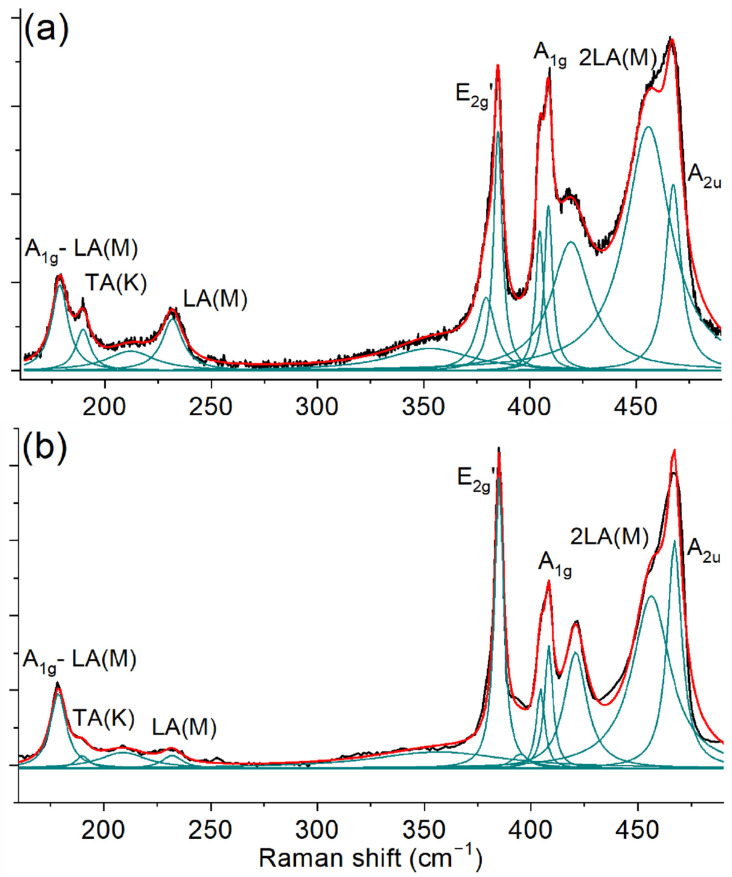
Raman spectra of MoS_2_: (**a**) Sulfurized MoO_3_ film; (**b**) exfoliated reference flake.

The spectroscopic Raman analysis of the obtained MoS_2_ film and exfoliated reference flake (Figure 5) was carried out on the resonant excitation wavelength (632.8 nm) corresponding to the direct bandgap (1.96 eV). Under these conditions, the obtained spectra contain first-order peaks denoted as E_2g_^1^ (Г) and A_1g_ (Г) corresponding to oscillatory modes inside the S-Mo-S layer in the parallel and perpendicular directions, but also overtones and combination peaks. For the sulfurized film, the difference between A_1g_ and E_2g_^1^ peaks was found to be approximately 23.7 cm^−1^, corresponding to the multilayer [49] MoS_2_, which is consistent with XPS thickness estimation. On the reference flake spectrum, the difference between these modes is 23.2 cm^−1^, which corresponds to three MoS_2_ monolayers.

The most significant differences between the discussed spectra are observed in the range from approximately 180 cm^−1^ to approximately 250 cm^−1^ containing several peaks (TA(K) ≈ 182 cm^−1^, LA(M) ≈ 227 cm^−1^), which are sensitive to lattice defects [50]. These modes cannot be observed in the spectra of the perfect MoS_2_ crystal structure, according to the momentum conservation rule. However, it can be satisfied by the phonon scattering from a defect, enabling the observation of zone-edge modes by Raman measurement [51]. The relative LA(M)/E_2g_^1^ (Г) intensity ratio was found to be approximately 5 times higher for the sulfurized film compared to the flake, which indicates a higher concentration of defects. For microcrystalline MoS_2_ films, the main type of defects is usually the crystalline domain boundaries. Therefore, the relative intensity of the LA(M) peak correlates with the size of the domains in accordance with earlier reports [28]. In general, the spectrum of the sulfurized film is typical for the structure containing sub-µm grains [52].

**Figure 6:**
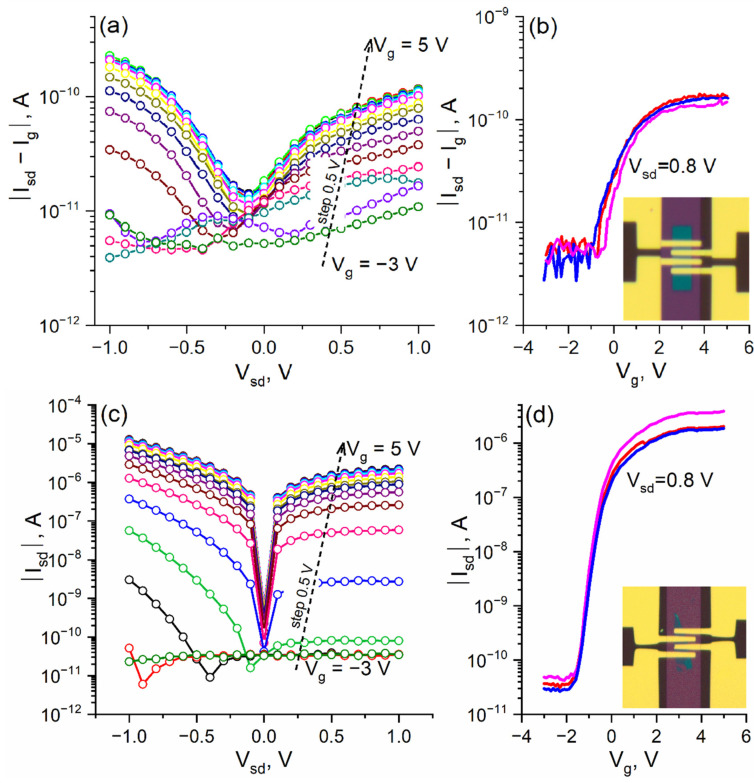
DC-IV source-drain characteristics of FETs based on sulfurized (**a**) and flake (**c**) MoS_2_ with a channel length of 1 µm measured at different gate voltages; transfer characteristics (**b**,**d**) of mentioned FETs (different colors correspond to nominally identical structures) measured at V_sd_ = 0.8 V (inset: Optical image of the FET).

After the basic MoS_2_ film characterization, four field-effect transistor devices with MoS_2_ channels were fabricated and investigated. Three structures were based on the films obtained via sulfurization, while the reference one was based on the exfoliated flake. DC-IV source-drain characteristics measured at different gate voltages are presented in Figure 6a,c for sulfurized and flake-based devices, respectively. 

All observed FET characteristics (Figure 6a,c) indicate the *n*-type MoS_2_ behavior since applying positive gate voltage resulted in a visible increase in channel conductivity. However, in the case of sulfurized MoS_2_ films, the applied gate voltage was strongly limited by leakages due to the high FET structure resistivity. As a result, the maximum achieved current modulation was found to be ≈40 (Figure 6b), which is comparable to the existing literature data [36,40,44]. For instance, Schram et al. reported a small ambipolar response with a weak (I_on_/I_off_ ≈ 3) gate modulation for CVD-grown WS_2_ nanolayers [40]. In contrast, plasma-enhanced ALD for WS_2_ growth with a technique of seed density inhibition for the larger crystalline size achievement resulted in a significant (up to 10^3^ times) modulation improvement. The authors ascribed such a difference to the specifics of CVD growth, namely, the surface-mediated reaction of the WF_6_ precursor with a sacrificial Si layer, which limited the crystal grain size and orientation. Similar microcrystalline MoS_2_ layers obtained by sputtering with additional sulfur powder annealing showed an I_on_/I_off_ ratio ≈ 30, which was limited by the remaining parasitic resistance [44]. The experiments of *Matsuura* et al. also revealed the necessity of sulfur vacancy healing to obtain any field effect. Despite this observation, here we reasonably guess that the sulfurization technique can hardly yield a significant sulfur deficiency (Figure 3). To date, the most prominent study of such films was carried out by Mahlouji et al., where thickness scaling and contact engineering were both considered [36,46]. It was found that the modulation magnitude has a strong maximum (I_on_/I_Off_ ≈ 2 × 10^4^) at a film thickness of ≈1.2 nm. Despite the fact that the observed discontinuity of such a thin film resulted in the presence of side contacts to MoS_2_, which has practical merit, its repeatability may be quite challenging. Furthermore, the contact engineering results were found to be not invariant in relation to a film thickness making further studies necessary.

In comparison, the electrical characteristics of the flake-based FET were found to be significantly superior, namely, it demonstrated four orders of magnitude greater I_on_ current (Figure 6d), which made leakage current contribution negligible (I_on_/I_off_ ~ 5 × 10^4^). According to the extracted subthreshold slope (SS) (320 mV/dec), field effect mobility was found to be ~0.4 cm^2^/V·s. The key extracted performance parameters for FET structures are summarized in Table 1.

In order to reveal the basic reason for the observed difference between the discussed films, additional TLM measurements, allowing us to separate the intrinsic MoS_2_ resistance and the contact resistance, were carried out. 

**Figure 7:**
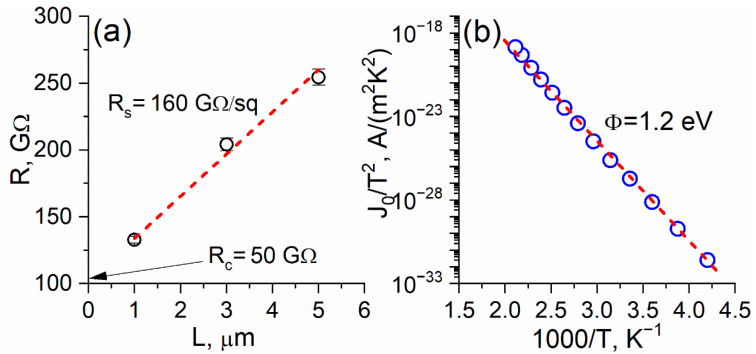
(**a**) Sulfurized MoS_2_-based FET resistivity as a function of channel length; (**b**) Arrhenius plot of J_0_/T^2^ as a function of 1/T used for calculating the effective Schottky barrier height using the thermionic emission model.

It was found that sulfurized MoS_2_-based FET resistivity linearly depends on the channel length. The corresponding sheet resistance of the MoS_2_ film was found to be ≈160 GΩ/sq (Figure 7a). As it has already been demonstrated, grain boundaries affect the electrical properties of polycrystalline MoS_2_ films significantly [12,53,54]. This typically manifests in a strong correlation between the carrier mobility and the I_On_/I_Off_ ratio and line defect density. Therefore, we can conclude that the intrinsic conductivity of the investigated MoS_2_ films is inferior to those reported in the literature [36,46] partially due to the relatively small grain size (Figure 4b) and their misorientation. In particular, the MoS_2_-based FET performance can be described by the normalized mean I_on_ current. For instance, the I_on_ current for a multiple-layer exfoliated MoS_2_ transistor was reported to be ~10^3^ µA/µm [55,56]. Simultaneously, microcrystalline MoS_2_ films showed significantly lower values—down to ≈10 µA/µm [36], which may be strongly governed by the source/drain-MoS_2_ contact quality [46]. Despite the fact that, in these works, the contribution of MoS_2_ resistance itself was not evaluated, it should be noted that the achieved I_on_ current was ~10^5^ times higher than in our case. 

However, the main factor limiting the sulfurized MoS_2_-based FETs performance was the contact resistivity (ρ_c_). According to Figure 6a, the ρ_c_ value was found to be approximately 4 × 10^3^ Ω·cm^2^ indicating the presence of a high Schottky barrier at the MoS_2_/Au interface [57,58]. The barrier height was calculated from the Arrhenius plot slope (Figure 7b) [59] and the result was ≈1.2 eV. The key role of contact resistance was further supported by the analysis of the reference device characteristics. The corresponding ρ_c_ value measured on the flake-based FET was found to be several orders of magnitude lower, which resulted in a barrier height comparable to the literature-reported value of ≈0.13 eV [60]. Therefore, in contrast to the natural MoS_2_ crystal, much more careful contact metal selection is necessary to demonstrate the full potential of the MoS_2_ film obtained via the sulfurization technique. Moreover, the MoS_2_ grain size increase is highly desired since it should naturally improve the mean current density. Previously, it was shown that the preliminary hydrogen treatment of the seed oxide film may significantly change the grain size distribution and reduce the WS_2_ resistivity [45]. Furthermore, the sulfurization temperature increase allowed the MoS_2_ grain size and orientation to vary between the edge-terminated (700 °C) and terrace-terminated structures (900 °C) [28]. Since, in both cases, two competing processes exist (oxide sublimation and sulfurization), changing the hydrogen treatment time and the sulfur supply parameters (start time, effective flow, and duration) may improve the resulting film performance. This may be a possible task for future research. Overall, such considerations confirm the necessity of detailed investigations into both contact and intrinsic properties of such microcrystalline material, since it might be of practical interest due to the fabrication simplicity and cost-effectiveness.

## 4. Conclusions

In this work, MoS_2_ films were obtained using a cost-effective method of sulfurization of thermal-ALD deposited MoO_3_. To estimate their performance, FET structures were fabricated and investigated, which demonstrated an I_on_/I_off_ ratio of ≈40. According to the experimental data, limiting factors were the polycrystalline structure and high contact resistance at the drain and source. These conclusions are supported by TEM grain size evaluation and Raman spectra featuring strong defect-attributed peaks as well as temperature-dependent conductivity behavior, which indicated a significant Schottky barrier influence on structure conductivity. The findings indicate the necessity of further careful development of fabrication processes including tuning sulfurization procedure parameters to increase grain size and careful contact metal selection based on the band structure investigations for films obtained via this technique. However, overall, due to its simplicity, cost-effectiveness, and low sensitivity to the substrate material, we still consider such a technique of MoS_2_ synthesis highly attractive for applications in next-generation electronics. The two-stage synthesis process described here may also be useful for the fabrication of other transition metal dichalcogenides ultrathin films (e.g., WS_2_).

## Figures and Tables

**Table 1:** FET structures performance parameters for sulfurized MoS_2_-based FETs and flake-based FETs.

Parameter	Sulfurized MoS_2_-Based FET	Flake-Based FET
I_on_/I_off_	40	5 × 10^4^
SS, mV/dec	2900	320
µ, cm^2^/V·s	4 × 10^−6^	0.4 cm^2^/V·s
Ion, A/µm	8 × 10^−11^	8 × 10^−6^

## Data Availability

Data are available at reasonable request from the authors.
